# Melphalan-based conditioning with post-transplant cyclophosphamide for peripheral blood stem cell transplantation: donor effect

**DOI:** 10.1038/s41409-025-02523-3

**Published:** 2025-02-27

**Authors:** Paul B. Koller, Tamer Othman, Dongyun Yang, Sally Mokhtari, Yazeed Samara, Amanda Blackmon, Vaibhav Agrawal, Hoda Pourhassan, Brian J. Ball, Idoroenyi Amanam, Shukaib Arslan, Salman Otoukesh, Karamjeet S. Sandhu, Ibrahim Aldoss, Haris Ali, Amandeep Salhotra, Ahmed Aribi, Andrew Artz, Pamela S. Becker, Vinod Pullarkat, Forest Marc Stewart, Eileen P. Smith, Anthony Stein, Guido Marcucci, Stephen J. Forman, Ryotaro Nakamura, Monzr M. Al Malki

**Affiliations:** 1 Department of Hematology and Hematopoietic Cell Transplantation, City of Hope National Medical Center, Duarte, CA USA; 2 Computational and Quantitative Medicine, City of Hope National Medical Center, Duarte, CA USA; 3 Clinical and Translational Project Development, City of Hope National Medical Center, Duarte, CA USA

**Keywords:** Preventive medicine, Stem cells

## Abstract

Fludarabine and melphalan (FM) conditioning offers effective disease control with an acceptable toxicity profile. Post-transplant cyclophosphamide (PTCy) for graft-versus-host disease (GVHD) prophylaxis has improved transplant outcomes. We retrospectively reviewed patients receiving FM-based transplants with PTCy at City of Hope. Of 248 patients included, 89 (35.9%) received hematopoietic cell transplant (HCT) from a matched related/unrelated donor (MRD/MUD), 118 (47.6%) from a haploidentical (HID) donor, and 49 (19.8%) from a mismatched unrelated donor (MMUD). There were no differences in acute and chronic GVHD based on donor type. The 2-year overall survival (OS) for patients receiving HID, MMUD, and MRD/MUD was 58%, 55%, and 70%; disease-free survival (DFS) was 52%, 48%, and 66%; and graft-versus-host/relapse-free survival (GRFS) were 48%, 40%, and 59%, respectively. OS, DFS, and GRFS were similar regardless of donor type on multivariable analysis. However, donor age ≥35 years was associated with lower OS and GRFS and higher 2-year non-relapse mortality (NRM) on multivariable analysis across all patients, regardless of donor type. FM with PTCy appears to produce similar outcomes between MRD/MUD, MMUD, and HID when adjusting for donors <35 years, and donor age seems to be the most important factor when selecting a donor with this regimen.

## Introduction

Reduced-intensity conditioning (RIC) has significantly increased accessibility to allogeneic hematopoietic cell transplantation (HCT) for older and less medically fit patients with hematologic malignancies [1, 2]. A retrospective analysis by the Center for International Blood and Marrow Transplant Research (CIMBTR) demonstrated that fludarabine/melphalan (FM) offers superior disease control compared to fludarabine/busulfan (FB), albeit with higher non-relapse mortality (NRM). This increased NRM may be driven in part by higher rates of acute and chronic graft-vs-host disease (GVHD) associated with FM [3]. FM conditioning leads to higher acute GVHD, and when combined with T-cell replete transplants, results in higher chronic GVHD than FB.

Post-transplant cyclophosphamide (PTCy) has improved HCT outcomes across different HLA disparities, including haploidentical (HID) and mismatched unrelated donors (MMUD), thus increasing HCT accessibility, particularly for patients from ethnic and racial minorities without a readily available matched donor [4–7]. Recently, in a phase 3 randomized control trial, PTCy combined with tacrolimus and mycophenolate mofetil improved 1-year GVHD/relapse-free survival (GRFS) compared to tacrolimus and methotrexate in matched donor RIC HCT [8]. However, most registry data comparing FM and FB involve patients receiving “conventional GVHD prophylaxis” with calcineurin inhibitor (CNI) based therapy; thus, no data is available that exclusively examines HCT outcomes using FM with the potentially new standard of care, PTCy-based GVHD prophylaxis.

The outcomes of patients undergoing HCT with PTCy using HID have previously been reported to be inferior to those using matched unrelated donors (MUD), particularly with RIC, although detailed information on causes of death were sparse due to the limitations of registry data [9]. Additionally, there is limited data comparing HID and MMUD using PTCy as GVHD prophylaxis. In a preliminary report from CIBMTR, no difference in outcomes was observed between HID and MMUD, but the small sample size and short follow-up period limits interpretation of these results [5]. In a second study conducted by the Acute Leukemia Working group of the European Society for Blood and Marrow Transplantation (EBMT) comparing HID and MMUD, the investigators reported no difference in cumulative incidence of relapse (CIR) and GRFS, but lower leukemia-free survival (LFS) and overall survival (OS) in the HID group. Of note, the HID group contained both bone marrow (BM) and peripheral blood stem cell (PBSC) grafts. Herein, we report a large single-center experience utilizing FM followed by PTCy-based GVHD prophylaxis using exclusively PBSC HCT. This study describes the outcomes of this RIC and GVHD prophylaxis combination across different donor types.

## Methods

### Study design, participants, and procedures

Adult patients who underwent PBSC HCT with FM followed by PTCy-based GVHD prophylaxis for a hematologic disorder at COH between January 2015-December 2021 were included in this retrospective analysis. Pediatric patients and those not receiving HCT at COH were excluded from the analysis. All donor types were allowed. MUD was defined as an 8/8 match (i.e., matched at HLA-A, -B, -C, and -DRB1). Detailed patient demographics and HCT information was obtained through retrospective chart review. The COH Institutional Review Board approved the study.

Patients with an MRD/MUD or MMUD received fludarabine 25 mg/m^2^ on days −8 to −4 (total 125 mg/m^2^) and melphalan 100 mg/m^2^ or 140 mg/m^2^ (if ≤55 years old) on day −3. Patients with a HID received melphalan on day −6, fludarabine 40 mg/m^2^ (total 160 mg/m^2^) on days −5 to −2, and TBI 200 cGy on day −1. Post-transplant cyclophosphamide 50 mg/kg was given on days +3 and +4. Tacrolimus, or sirolimus in patients unable to receive tacrolimus, was started on day +5 and dose-adjusted to maintain trough level of 5–10 [10], and was converted to oral on discharge (in the case of tacrolimus), and slowly tapered over 6 months starting on day +90 in the absence of GVHD. Mycophenolate mofetil was given at 15 mg/kg (capped at 1 g) three times daily starting on day +5 and was stopped on day +35 in the absence of GVHD.

Non-emergent use of corticosteroids for emesis control or transfusion reactions were prohibited until day 5, but use of systemic steroids and other immunosuppressants like tocilizumab was allowed based on individual patient risk factors suggesting a poor prognosis and the patient’s overall clinical status as per the discretion of the treating physician. Hematologic support using formulary granulocyte colony stimulating factor (G-CSF) 5 µg/kg/day was initiated on day 5 and was administered daily until absolute neutrophil count (ANC) reached ≥1.5 × 10^9^/L for 3 consecutive days as per institutional standards. Infection prophylaxis against bacterial, viral, and fungal (including *Pneumocystis jiroveci*) pathogens were initiated per institutional practice and in accordance with the American Society for Transplantation and Cellular Therapy (ASTCT) guidelines for post-HCT infection prevention [11]. Weekly cytomegalovirus (CMV) polymerase chain reaction (PCR) testing was performed to monitor for detectable levels requiring preemptive therapy. Letermovir was given for CMV prophylaxis to CMV seropositive patients upon FDA approval in November of 2017.

### Study outcomes and statistical analyses

Overall survival (OS) was the primary endpoint, which was defined as time HCT to death. Patients who remained alive were censored at their date of last follow-up. Secondary endpoints included disease-free survival (DFS), defined as time from allogeneic HCT to disease relapse or death. Patients alive without evidence of disease at study cutoff were censored at their date of last follow-up. Other secondary endpoints include non-relapse mortality (NRM), defined as death without prior relapse of their disease post-HCT, CIR, grades II-IV and III-IV acute GVHD per the consensus criteria [12], moderate/severe chronic GVHD as per NIH criteria [13], GRFS, defined as time from allogeneic HCT to grades II-IV acute GVHD or moderate/severe chronic GVHD, disease relapse or death, and neutrophil and platelet engraftment.

Baseline patient demographic, disease, and transplant characteristics were summarized using descriptive statistics and were compared using the log-rank test. Kaplan–Meier Curves and log-rank tests were used to calculate and compare OS and DFS, respectively. CIR and NRM and GVHD were calculated and compared via a competing-risk analysis and Gray’s test, respectively. Univariate and multivariate analyses (UVA and MVA) were performed using the multivariable Cox regression model for OS and DFS, and multivariable Fine and Gray regression model for the other variables. The primary aim was to evaluate the effect of donor types on HCT outcomes. All analyses were performed using SAS version 9.4 (SAS Institute, Cary, NC). All tests were 2 sided at a significance level of 0.05.

## Results

### Patient, disease, and transplant characteristics

A total of 248 patients were included in this study. Their baseline characteristics are summarized in Table 1. Median recipient age was 63 years (range, 20–82), and 63.7% of the patients were male. Karnofsky performance status (KPS) was ≤80% in more than third of patients. Almost half of the patients (47.6%) had a HCT comorbidity index of ≥3. The most common diagnoses among the patients were acute myeloid leukemia (AML) (*n* = 90), acute lymphoblastic leukemia (ALL) (*n* = 39), myelodysplastic syndrome/myeloproliferative neoplasms (MDS/MPN) (*n* = 58), and lymphoma (*n* = 40). The median donor age was 32 years (range, 11–65). Donors were MRD/MUD in 32.7%, HID in 47.6% and MMUD in 19.8% of cases.

**Table 1:** Baseline patient, disease, and transplant characteristics.

	MRD/MUD (*N* = 81)	MMUD (*N* = 49)	HID (*N* = 118)	Total (*N* = 248)	*P* value
Age at HCT, years					0.004
Median	65	63	61	63	
Interquartile range	60, 70	58, 66	54, 66	57, 67	
Range	(22–82)	(33–75)	(20–76)	(20–82)	
Recipient sex					0.064
Male	55 (67.9%)	24 (49%)	79 (66.9%)	158 (63.7%)	
Female	26 (32.1%)	25 (51%)	39 (33.1%)	90 (36.3%)	
Karnofsky performance status %					0.23
90–100	58 (71.6%)	32 (65.3%)	67 (56.8%)	157 (63.3%)	
80	19 (23.5%)	14 (28.6%)	37 (31.4%)	70 (28.2%)	
≤70	4 (4.9%)	3 (6.1%)	14 (11.9%)	21 (8.5%)	
HCT comorbidity index					0.32
0	13 (16%)	10 (20.4%)	32 (27.1%)	55 (22.2%)	
1-2	25 (30.9%)	13 (26.5%)	37 (31.4%)	75 (30.2%)	
≥3	43 (53.1%)	26 (53.1%)	49 (41.5%)	118 (47.6%)	
Primary diagnosis					0.22
AML	37 (45.7%)	13 (26.5%)	40 (33.9%)	90 (36.3%)	
ALL	13 (16%)	11 (22.4%)	15 (12.7%)	39 (15.7%)	
MDS/MPN	18 (22.2%)	13 (26.5%)	27 (22.9%)	58 (23.4%)	
Lymphoma	10 (12.3%)	7 (14.3%)	23 (19.5%)	40 (16.1%)	
CML/CMMoL	2 (2.5%)	2 (4.1%)	10 (8.5%)	14 (5.6%)	
Others	1 (1.2%)	3 (6.1%)	3 (2.5%)	7 (2.8%)	
DRI					0.010
Low/intermediate	70 (86.4%)	33 (67.3%)	83 (70.3%)	186 (75%)	
High/very high	11 (13.6%)	16 (32.7%)	35 (29.7%)	62 (25%)	
Female donor to male recipient					0.44
Yes	17 (21%)	6 (12.2%)	20 (16.9%)	43 (17.3%)	
No	64 (79%)	43 (87.8%)	98 (83.1%)	205 (82.7%)	
Donor age					0.12
Median	29	32	34	32	
Interquartile range	25, 37	26, 38	25, 42	25, 39	
Range	(19–64)	(19–55)	(11–65)	(11–65)	
ABO blood group compatibility					0.094
ABO compatible	44 (54.3%)	20 (40.8%)	76 (64.4%)	140 (56.5%)	
Minor mismatch (donor is O)	10 (12.3%)	7 (14.3%)	16 (13.6%)	33 (13.3%)	
Major mismatch (Recipient is O)	18 (22.2%)	14 (28.6%)	15 (12.7%)	47 (19%)	
Bidirectional (None are O)	9 (11.1%)	8 (16.3%)	11 (9.3%)	28 (11.3%)	
Donor/Recipient CMV serostatus					<0.001
D−/R−	20 (24.7%)	6 (12.2%)	10 (8.5%)	36 (14.5%)	
D−/R+	29 (35.8%)	23 (46.9%)	31 (26.3%)	83 (33.5%)	
D+/R−	9 (11.1%)	2 (4.1%)	8 (6.8%)	19 (7.7%)	
D+/R+	23 (28.4%)	18 (36.7%)	68 (57.6%)	109 (44%)	
Unknown	0 (0%)	0 (0%)	1 (0.8%)	1 (0.4%)	
Conditioning regimen					
FLUDARABINE/MELPHALAN	80 (98.8%)	49 (100%)		129 (52%)	
FLUDARABINE/MELPHALAN/TBI	1 (1.2%)		118 (100%)	119 (48%)	
GVHD prophylaxis					
CTX/SIROLIMUS/CELLCEPT	15 (18.5%)	0 (0%)	2 (1.7%)	17 (6.9%)	
CTX/TACROLIMUS/CELLCEPT	66 (81.5%)	49 (100%)	116 (98.3%)	231 (93.1%)	
HCT period					<0.001
2015–2017	3 (3.7%)	11 (22.4%)	33 (28%)	47 (19%)	
2018–2021	78 (96.3%)	38 (77.6%)	85 (72%)	201 (81%)	

### Engraftment and GVHD

The median time to neutrophil engraftment was 18 days (range, 18–19), and the median time to platelet engraftment was 32 days (range, 30–34). Multivariate analysis (MVA) revealed no significant difference in neutrophil engraftment by day 28 based on donor type. However, recipients of MRD/MUD showed a higher rate of platelet engraftment by day 35 (*P* = 0.020) compared to those receiving grafts from HID and MMUD (Supplementary Table 1). There were no significant differences in engraftment rates based on patient age or donor age.

The cumulative incidence of grade II-IV acute GVHD by day 100 was 39.5% (95% CI, 33.4–45.6), and grade III-IV acute GVHD was 14.5% (95% CI, 10.5–19.2). The 1-year cumulative incidence of extensive chronic GVHD was 27.4% (95% CI, 22.0–33.1) for all patients. MVA revealed no significant differences in the incidence of acute or chronic GVHD based on donor type.

### OS, DFS, and GRFS

**Fig. 1:**
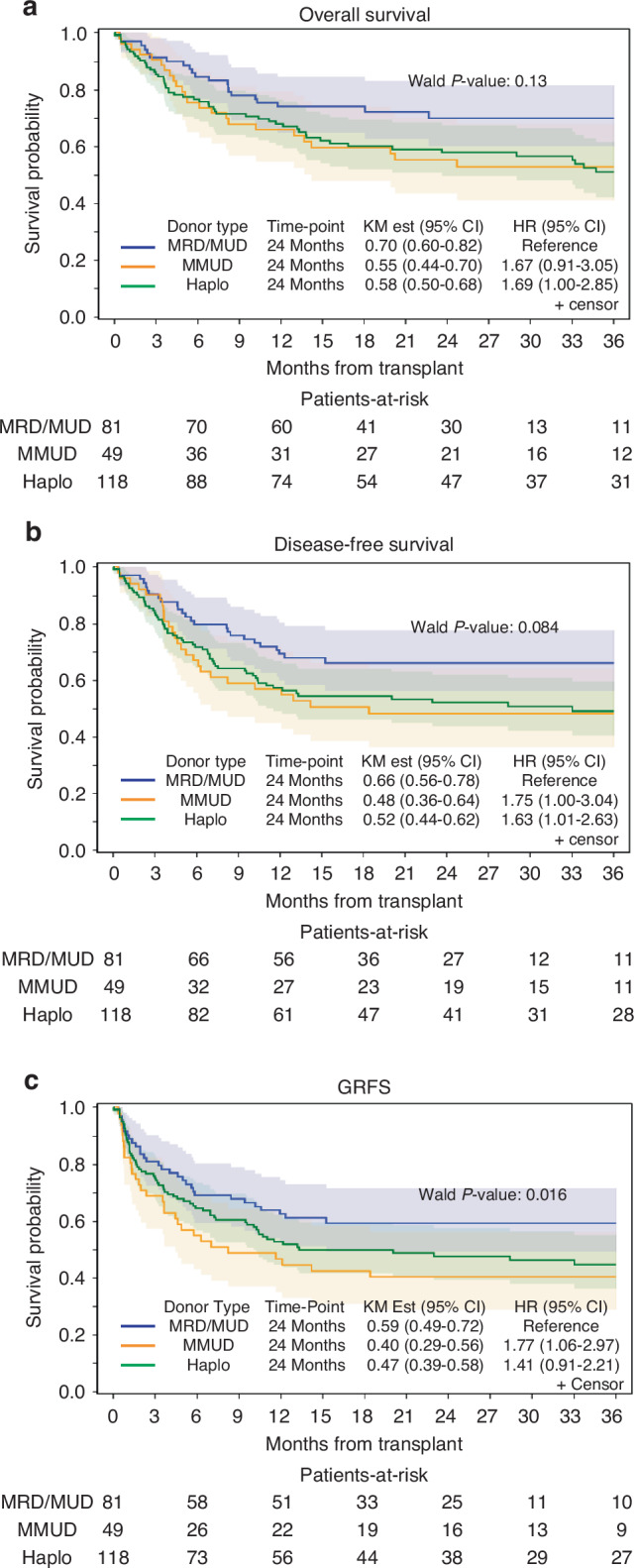
Adjusted survival figures based on donor type. Adjusted overall survival based on donor type (**a**), disease-free survival based on donor type (**b**), graft-vs-host/relapse-free survival based on donor type (**c**).

With a median follow-up for surviving patients of 24.4 months (range, 3.3–81.2), the 2-year OS, DFS, and GRFS for all patients were 60.4% (95% CI, 53.7–66.5), 55.5% (95% CI, 48.9–61.6), and 49.2% (95% CI, 43.2–56.0), respectively. The adjusted 2-year OS were 58% for HID, 55% for MMUD, and 70% for MRD/MUD (*P* = 0.13), the adjusted 2-year DFS rates were 52% for HID, 48% for MMUD, and 66% for MRD/MUD (*P* = 0.084), and the GRFS rates were 48% for HID, 40% for MMUD, and 59% for MRD/MUD (*P* = 0.088) (Fig. 1a–c).

Lower HCT comorbidity index, low/intermediate DRI, and HCT during the recent era (2018–2021) were all associated with improved OS on MVA. Additionally, a low/intermediate DRI was linked to improved DFS and GRFS. Importantly, MVA revealed no significant differences in OS, DFS, and GRFS based on donor type, indicating that outcomes were comparable across MRD/MUD, MMUD, and HID when adjusting for other factors.

### CIR and NRM

**Fig. 2:**
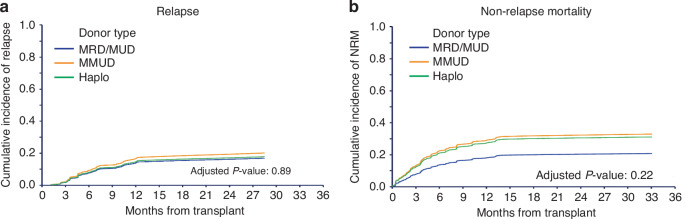
Adjusted competing risk curves based on donor type. Adjusted non-relapse mortality based on donor type (**a**), and cumulative incidence of relapse based on donor type (**b**).

The 2-year CIR and NRM for all patients were 17.2% (95% CI, 13.0–22.8) and 27.7% (95% CI, 22.5–34.0), respectively. A high or very-high disease risk index (DRI) was significantly associated with a higher CIR (*P* < 0.001). NRM was notably lower in patients with KPS of ≥90% (*P* = 0.049). There was no significant difference in NRM based on the HCT comorbidity index. On MVA donor type had no impact on CIR or NRM (Fig. 2a, b). Table 2 summarizes the causes of death across the entire cohort and by donor type group. Overall, 62.7% of patients died from NRM, while 37.3% succumbed to disease relapse.

**Table 2:** Causes of death.

Cause of death	Matched (*N* = 28)	MMUD (*N* = 25)	HID (*N* = 57)	Total (*N* = 110)	*p* value
CRS	0 (0%)	0 (0%)	1 (1.8%)	1 (0.9%)	0.93
GVHD	5 (17.9%)	6 (24%)	9 (15.8%)	20 (18.2%)	
Infection	6 (21.4%)	8 (32%)	20 (35.1%)	34 (30.9%)	
Organ dysfunction	3 (10.7%)	2 (8%)	7 (12.3%)	12 (10.9%)	
Relapse	14 (50%)	9 (36%)	18 (31.6%)	41 (37.3%)	
Unknown	0 (0%)	0 (0%)	1 (1.8%)	1 (0.9%)	
VOD	0 (0%)	0 (0%)	1 (1.8%)	1 (0.9%)	

### Impact of donor type and age on outcomes

**Fig. 3:**
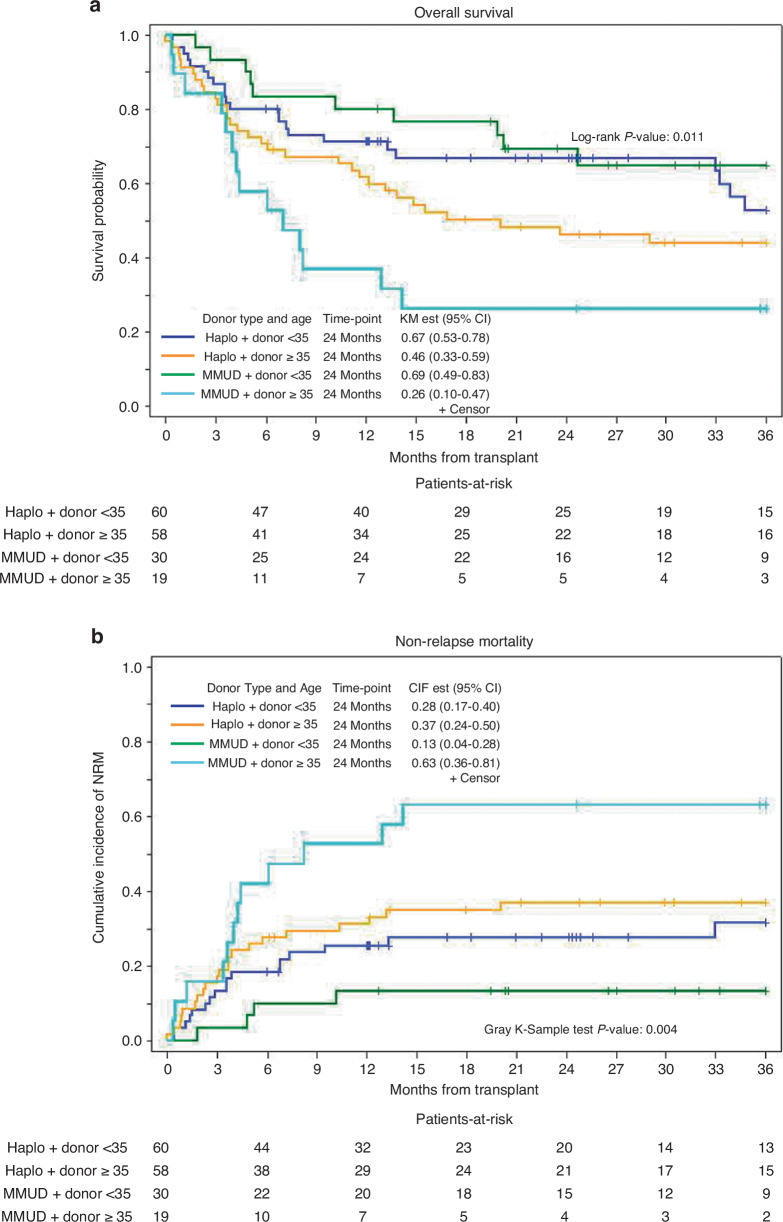
Outcomes based on donor type and age. Overall survival based on donor type and age (**a**). Non-relapse mortality based on donor type and age (**b**).

Figure 3a, b shows improvement in OS and NRM in patients with donors <35 years of age, regardless of whether a HID and MMUD was utilized. Donor age ≥35 years was associated with inferior OS (*P* = 0.015) and GRFS (*P* = 0.043) (Supplementary Table 1). While matched donors showed improved OS, DFS, and GRFS on UVA, these differences were not significant on MVA. When comparing baseline characteristics, there were significantly more matched donors in the younger donor cohort (<35 years) (*P* = 0.021, Supplementary Table 2). There were no differences in incidence or grade of hemorrhagic cystitis between the donor groups (Supplementary Table 3). We observed a higher incidence of fungal and viral infections with HID, while there was no difference in bacterial infections between the two groups. Moreover, there was a higher incidence of BMT CTN grade 3 infections in patients with a mismatch donor (i.e., MMUD or haplo) compared to a matched donor (MRD/MUD), 3.7% vs 11.4% (*P* = 0.046).

Moreover, NRM was lower in patients with donors <35 years on both UVA (*P* = 0.008) and MVA (*P* = 0.028) However, there was no significant difference in NRM based on donor type on MVA. Similarly, MVA showed no differences in CIR based on donor type (*P* = 0.89) or age (*P* = 0.75). There were also no significant differences in the incidence of grade II-IV and grade III-IV acute GVHD, or in any chronic GVHD and extensive chronic GVHD, based on donor type or donor age.

## Discussion

We report our experience with FM conditioning combined with PTCy-based GVHD prophylaxis, demonstrating encouraging disease control and survival outcomes, with acceptable GVHD and NRM. The BMT CTN 1703 demonstrated a clear advantage in 1-year GRFS using PTCy for GVHD prophylaxis across various RIC regimens. However, it remains unclear how each conditioning regimen specifically interacts with PTCy, but further analyses are ongoing. To our knowledge, this is the first report exclusively investigating FM with PTCy-based GVHD prophylaxis using a PBSC graft source.

Previous studies have explored the use of HID and PTCy-based GVHD prophylaxis. Earlier studies predominately utilized BM as the graft source, and validated the use of PTCy with non-myeloablative (NMA) conditioning regimen, achieving acceptable survival rates and incidences of GVHD and graft failure [7, 14]. However, relapse rates were high, 51% within the first year, indicating the need for more effective conditioning regimens with haplo-based transplants. Subsequent studies have evaluated PTCy with a PBSC graft source and myeloablative conditioning (MAC) [15], RIC,^8^ and NMA conditioning regimens [16]. The BMT CTN 1703 trial reported a 1-year CIR of 20.8% with PTCy, higher than what we observed in our study. This may be due to the exclusive use of FM in our study, which provides excellent disease control [3]. Eastburg et al. conducted the only retrospective case series of 38 patients that investigated FM, a commonly utilized RIC regimen, with HID and PTCy-based GVHD prophylaxis. They reported a high NRM of 34% [17]. The relatively high NRM might be due to a significant number of patients with active disease at the time of transplant, as this has been shown to be a predictor of increased NRM with AML [18]. Additionally, TBI was not used in this study, which presumably can help eliminate pre-existing alloreactive T cells involved in rejection and cytokine release syndrome [19]. Solh et al. conducted a prospective phase 2 study enrolling 25 patients utilizing a HID with FM-conditioning and a PBSC graft and PTCy, showing similar outcomes to a contemporaneous cohort treated with NMA-based haplo-HCT with PTCy in terms of 2-year OS and DFS [20]. Our study includes a larger sample size and multiple donor types, allowing for a better description of differences across HLA disparities. We show that the regimen can be effective irrespective of the degree of HLA-matching after adjusting for donor age. Donor age <35 appears to be the most significant factor predictive of OS and GRFS by reducing NRM. This corroborates the findings of an EBMT study led by Sanz et al., which showed that patients receiving HCT from a donor younger than 30 years had less relapse, improved LFS, OS, and GRFS among patients with 9/10 MMUDs and 10/10 MUDs receiving PTCy [21]. A second study led by Sanz et al. showed that among patients with HIDs, age of donors older than 37 years negatively impacted NRM, LFS, and OS [22]. Additionally, we observed that OS improved over time in the recent era, likely reflecting improved experience and a learning curve with this regimen. Finally, patients with HIDs had a higher incidence of fungal and viral infections, and those with a mismatch donor had a higher incidence of more severe infections. This could be in part due to a higher degree of T-cell dysregulation that is related to HLA mismatch [23].

Limitations of our study include its retrospective nature, single-center analysis, and relatively small sample size, particularly for the MMUD cohort. Additionally, certain factors that could impact outcomes, such as the presence of donor-specific antibodies, use of cryopreserved versus fresh cells, and donor availability, were not incorporated into this analysis. Despite these limitations, our results are significant as they show that FM combined with PTCy-based GVHD prophylaxis and a PBSC graft source can lead to promising disease and GVHD control and survival with acceptable NRM, regardless of donor source. This is particularly significant for patients who are racially and ethnically diverse, with lower probability of finding matched donor in the registry [5]. Future studies are needed to confirm that these findings can be reproduced in a prospective and multicenter trial properly powered to detect equal outcomes between a MMUD and HID with this regimen, and to study interventions aimed at reducing NRM.

To conclude, FM with PTCy was associated with promising disease control and acceptable NRM. After adjusting for donor age, there was no significant difference in OS and GRFS based on donor type (i.e., HID vs. MMUD vs. MRD/MUD). Donors <35 years old were the strongest predictor of improved outcomes and appear to be the most important factor to consider when selecting a donor for FM with PTCy and a PBSC source.

## Supplementary information

Supplementary Table 1

Supplementary Table 2

Supplementary Table 3

## Data Availability

Raw data used to generate these results are available upon request. Please contact the corresponding author.
