# Supplementary material for: Melphalan-based conditioning with post-transplant cyclophosphamide for peripheral blood stem cell transplantation: donor effect
Source: Bone Marrow Transplant. 2025 Feb 27;60(5):625–31. doi: 10.1038/s41409-025-02523-3 (PMC12061766; doi:10.1038/s41409-025-02523-3)
Supplement: Supplementary file 1 — Supplementary Table 1 [file 41409_2025_2523_MOESM1_ESM.docx]

**Supplemental Table 1:** Univariate and multivariate analyses

|  | | | *Overall Survival* | | | | | *DFS* | | | | | *GRFS* | | | | |
| --- | --- | --- | --- | --- | --- | --- | --- | --- | --- | --- | --- | --- | --- | --- | --- | --- | --- |
|  |  | *N* | *2 Yr (95%CI)** | *HR (95%CI)** | *P** | *Adjusted HR (95%CI)* † | *Wald test P*† | *2 Yr (95%CI)** | *HR (95%CI)** | *P** | *Adjusted HR (95%CI)* † | *Wald test P*† | *1 Yr (95%CI)** | *HR (95%CI)** | *P** | *Adjusted HR (95%CI)* † | *Wald test P*† |
| Age, yrs | <70 | 211 | 0.617(0.544,0.681) | Reference | 0.68 | Reference | 0.57 | 0.559(0.487,0.625) | Reference | 0.65 | Reference | 0.27 | 0.543(0.473,0.608) | Reference | 0.77 | Reference | 0.87 |
|  | ≥70 | 37 | 0.527(0.340,0.683) | 1.12(0.65,1.91) |  | 1.17(0.68,2.02) |  | 0.505(0.333,0.654) | 1.13(0.68,1.87) |  | 1.34(0.80,2.24) |  | 0.541(0.369,0.684) | 0.93(0.57,1.52) |  | 1.04(0.63,1.72) |  |
| Sex | M | 158 | 0.608(0.525,0.681) | Reference | 0.95 | Reference | 0.82 | 0.536(0.454,0.611) | Reference | 0.38 | Reference | 0.34 | 0.512(0.431,0.586) | Reference | 0.51 | Reference | 0.49 |
|  | F | 90 | 0.592(0.472,0.693) | 0.99(0.66,1.48) |  | 1.05(0.69,1.60) |  | 0.572(0.455,0.672) | 0.84(0.57,1.25) |  | 0.82(0.55,1.23) |  | 0.598(0.489,0.691) | 0.88(0.61,1.28) |  | 0.88(0.60,1.28) |  |
| KPS | ≥90 | 157 | 0.640(0.556,0.712) | Reference | 0.055 | Reference | 0.26 | 0.583(0.500,0.657) | Reference | 0.20 | Reference | 0.40 | 0.547(0.465,0.621) | Reference | 0.50 | Reference | 0.67 |
|  | 80 | 70 | 0.590(0.458,0.700) | 1.29(0.83,2.00) |  | 1.30(0.83,2.02) |  | 0.524(0.397,0.637) | 1.18(0.77,1.79) |  | 1.11(0.73,1.70) |  | 0.555(0.431,0.663) | 1.07(0.72,1.59) |  | 1.03(0.69,1.53) |  |
|  | ≤70 | 21 | 0.368(0.153,0.588) | 2.07(1.11,3.86) |  | 1.62(0.84,3.14) |  | 0.390(0.173,0.603) | 1.73(0.93,3.20) |  | 1.52(0.82,2.83) |  | 0.471(0.251,0.664) | 1.44(0.78,2.64) |  | 1.32(0.72,2.44) |  |
| HCTCI | 0 | 55 | 0.736(0.595,0.835) | Reference | 0.060 | Reference | **0.033** | 0.635(0.486,0.751) | Reference | 0.17 | Reference | 0.12 | 0.655(0.513,0.764) | Reference | 0.48 | Reference | 0.42 |
|  | 1-2 | 75 | 0.545(0.419,0.656) | 2.03(1.11,3.72) |  | 2.25(1.22,4.16) |  | 0.463(0.344,0.574) | 1.66(0.97,2.85) |  | 1.77(1.03,3.06) |  | 0.477(0.360,0.584) | 1.35(0.83,2.22) |  | 1.40(0.84,2.31) |  |
|  | ≥3 | 118 | 0.579(0.480,0.666) | 1.78(1.00,3.17) |  | 1.90(1.06,3.40) |  | 0.566(0.470,0.652) | 1.34(0.80,2.24) |  | 1.46(0.86,2.47) |  | 0.532(0.438,0.617) | 1.20(0.75,1.91) |  | 1.29(0.80,2.08) |  |
| DRI | Low-int | 186 | 0.651(0.574,0.717) | Reference | **0.008** | Reference | **0.015** | 0.609(0.533,0.676) | Reference | **<0.001** | Reference | **0.003** | 0.595(0.521,0.662) | Reference | **<0.001** | Reference | **0.002** |
|  | H/VH | 62 | 0.463(0.328,0.588) | 1.73(1.15,2.62) |  | 1.68(1.11,2.56) |  | 0.378(0.252,0.502) | 1.91(1.29,2.83) |  | 1.82(1.22,2.71) |  | 0.387(0.267,0.505) | 1.88(1.30,2.72) |  | 1.79(1.23,2.61) |  |
| F to M | No | 205 | 0.607(0.532,0.673) | Reference | 0.97 | Reference | 0.89 | 0.559(0.486,0.627) | Reference | 0.54 | Reference | 0.38 | 0.565(0.494,0.630) | Reference | 0.57 | Reference | 0.47 |
|  | Yes | 43 | 0.594(0.429,0.725) | 1.01(0.60,1.70) |  | 1.04(0.61,1.77) |  | 0.505(0.347,0.643) | 1.16(0.72,1.87) |  | 1.24(0.77,2.01) |  | 0.434(0.283,0.576) | 1.14(0.73,1.78) |  | 1.18(0.75,1.85) |  |
| Dr age | ≤34 | 147 | 0.682(0.596,0.754) | Reference | **0.018** | Reference | **0.015** | 0.611(0.526,0.686) | Reference | **0.044** | Reference | 0.057 | 0.584(0.500,0.659) | Reference | **0.043** | Reference | **0.043** |
|  | ≥35 | 101 | 0.494(0.389,0.590) | 1.60(1.08,2.36) |  | 1.64(1.10,2.44) |  | 0.468(0.365,0.564) | 1.46(1.01,2.13) |  | 1.45(0.99,2.11) |  | 0.482(0.381,0.575) | 1.43(1.01,2.04) |  | 1.45(1.01,2.08) |  |
| HCT era | 2015-17 | 47 | 0.447(0.302,0.581) | Reference | **0.008** | Reference | **0.011** | 0.404(0.265,0.539) | Reference | **0.010** | Reference | 0.17 | 0.404(0.265,0.539) | Reference | **0.034** | Reference | 0.29 |
|  | 2018-21 | 201 | 0.641(0.566,0.707) | 0.56(0.36,0.88) |  | 0.56(0.36,0.88) |  | 0.583(0.509,0.651) | 0.58(0.37,0.89) |  | 0.73(0.47,1.14) |  | 0.575(0.503,0.640) | 0.64(0.42,0.97) |  | 0.79(0.52,1.22) |  |
| Donor | M | 81 | 0.725(0.606,0.813) | Reference | **0.024** | Reference | 0.13 | 0.684(0.568,0.775) | Reference | **0.016** | Reference | 0.084 | 0.654(0.540,0.747) | Reference | **0.016** | Reference | 0.088 |
|  | MM | 49 | 0.526(0.377,0.655) | 1.95(1.08,3.50) |  | 1.67(0.91,3.05) |  | 0.467(0.323,0.599) | 1.95(1.13,3.38) |  | 1.75(1.00,3.04) |  | 0.449(0.307,0.581) | 1.99(1.20,3.30) |  | 1.77(1.06,2.97) |  |
|  | Haplo | 118 | 0.561(0.462,0.648) | 1.92(1.16,3.18) |  | 1.69(1.00,2.85) |  | 0.498(0.402,0.588) | 1.88(1.18,3.00) |  | 1.63(1.01,2.63) |  | 0.503(0.409,0.590) | 1.66(1.08,2.56) |  | 1.41(0.91,2.21) |  |

* Based on Kaplan-Meier curves and log-rank test.

† Based on multivariable Cox regression model. The model for OS was adjusted for HCTCI, DRI, donor age, and HCT era. The model for DFS was adjusted for DRI, donor age and donor type. The model for GRFS was adjusted for DRI, donor age, and donor type.

|  | | | *Relapse* | | | | | *NRM* | | | | | |
| --- | --- | --- | --- | --- | --- | --- | --- | --- | --- | --- | --- | --- | --- |
|  |  | *N* | *2 Yr (95%CI)** | *HR (95%CI)** | *Gray P** | *Adjusted HR (95%CI)* † | *FG test P*† | *100 day (95%CI)** | *1 Yr (95%CI)** | *HR (95%CI)** | *Gray P** | *Adjusted HR (95%CI)* † | *FG test P*† |
| Age, yrs | <70 | 211 | 0.159(0.112,0.213) | Reference | 0.22 | Reference | 0.16 | 0.114(0.075,0.161) | 0.253(0.196,0.313) | Reference | 0.66 | Reference | 0.64 |
|  | ≥70 | 37 | 0.248(0.120,0.399) | 1.58(0.77,3.26) |  | 1.70(0.81,3.53) |  | 0.135(0.049,0.266) | 0.216(0.100,0.361) | 0.86(0.42,1.73) |  | 0.84(0.41,1.73) |  |
| Sex | M | 158 | 0.189(0.131,0.256) | Reference | 0.27 | Reference | 0.37 | 0.133(0.085,0.191) | 0.261(0.195,0.331) | Reference | 0.95 | Reference | 0.79 |
|  | F | 90 | 0.143(0.077,0.229) | 0.68(0.35,1.33) |  | 0.74(0.38,1.43) |  | 0.089(0.041,0.159) | 0.224(0.143,0.315) | 1.01(0.62,1.65) |  | 0.94(0.56,1.55) |  |
| KPS | ≥90 | 157 | 0.190(0.132,0.257) | Reference | 0.27 | Reference | 0.21 | 0.102(0.061,0.155) | 0.204(0.145,0.271) | Reference | **0.006** | Reference | **0.049** |
|  | 80 | 70 | 0.165(0.087,0.265) | 0.80(0.40,1.58) |  | 0.75(0.37,1.52) |  | 0.129(0.063,0.219) | 0.274(0.174,0.383) | 1.49(0.88,2.52) |  | 1.40(0.83,2.36) |  |
|  | ≤70 | 21 | 0.048(0.003,0.205) | 0.24(0.03,1.74) |  | 0.18(0.02,1.40) |  | 0.190(0.057,0.383) | 0.485(0.253,0.683) | 3.01(1.54,5.88) |  | 2.27(1.16,4.45) |  |
| HCTCI | 0 | 55 | 0.158(0.072,0.275) | Reference | 0.28 | Reference | 0.31 | 0.055(0.014,0.137) | 0.145(0.067,0.252) | Reference | 0.31 | Reference | 0.19 |
|  | 1-2 | 75 | 0.240(0.146,0.346) | 1.44(0.65,3.19) |  | 1.40(0.64,3.07) |  | 0.120(0.059,0.205) | 0.297(0.197,0.403) | 1.63(0.79,3.33) |  | 1.78(0.86,3.71) |  |
|  | ≥3 | 118 | 0.137(0.082,0.206) | 0.84(0.37,1.90) |  | 0.82(0.37,1.85) |  | 0.144(0.088,0.214) | 0.264(0.188,0.346) | 1.62(0.83,3.16) |  | 1.84(0.94,3.62) |  |
| DRI | Low-int | 186 | 0.122(0.079,0.175) | Reference | **<0.001** | Reference | **<0.001** | 0.118(0.077,0.169) | 0.243(0.184,0.307) | Reference | 0.54 | Reference | 0.54 |
|  | H/VH | 62 | 0.321(0.204,0.445) | 3.09(1.70,5.64) |  | 3.09(1.70,5.64) |  | 0.113(0.049,0.206) | 0.260(0.157,0.375) | 1.18(0.70,1.99) |  | 1.17(0.71,1.93) |  |
| F to M | No | 205 | 0.169(0.120,0.225) | Reference | 0.81 | Reference | 0.88 | 0.112(0.074,0.160) | 0.235(0.179,0.295) | Reference | 0.62 | Reference | 0.41 |
|  | Yes | 43 | 0.191(0.088,0.323) | 1.10(0.52,2.35) |  | 1.06(0.50,2.21) |  | 0.140(0.056,0.261) | 0.305(0.173,0.446) | 1.17(0.64,2.15) |  | 1.29(0.70,2.37) |  |
| Dr age | ≤34 | 147 | 0.182(0.123,0.249) | Reference | 0.62 | Reference | 0.75 | 0.095(0.055,0.149) | 0.198(0.138,0.267) | Reference | **0.008** | Reference | **0.028** |
|  | ≥35 | 101 | 0.156(0.091,0.237) | 0.86(0.47,1.59) |  | 0.91(0.49,1.67) |  | 0.149(0.087,0.225) | 0.318(0.229,0.411) | 1.89(1.18,3.05) |  | 1.72(1.06,2.77) |  |
| HCT era | 2015-17 | 47 | 0.170(0.078,0.292) | Reference | 0.89 | Reference | 0.46 | 0.191(0.094,0.316) | 0.404(0.263,0.541) | Reference | **0.010** | Reference | 0.098 |
|  | 2018-21 | 201 | 0.174(0.123,0.232) | 1.02(0.47,2.23) |  | 1.39(0.59,3.27) |  | 0.100(0.063,0.146) | 0.210(0.157,0.269) | 0.49(0.29,0.84) |  | 0.64(0.37,1.09) |  |
| Donor | M | 81 | 0.143(0.075,0.233) | Reference | 0.62 | Reference | 0.89 | 0.074(0.030,0.145) | 0.173(0.100,0.263) | Reference | **0.049** | Reference | 0.22 |
|  | MM | 49 | 0.205(0.104,0.329) | 1.53(0.65,3.61) |  | 1.23(0.52,2.88) |  | 0.082(0.026,0.180) | 0.286(0.167,0.417) | 2.00(0.99,4.06) |  | 1.75(0.87,3.55) |  |
|  | Haplo | 118 | 0.178(0.113,0.255) | 1.33(0.65,2.75) |  | 1.06(0.51,2.24) |  | 0.161(0.101,0.233) | 0.282(0.203,0.366) | 2.06(1.12,3.79) |  | 1.63(0.88,3.01) |  |

* Based on cumulative incidence curves and Gray’s test.

† Based on multivariable Fine and Gray regression model. The model for relapse was adjusted for DRI. The model for NRM was adjusted for KPS, donor age, and HCT era.

|  | | | *Neutrophil Engraftment* | | | | | *Platelet Engraftment* | | | | |
| --- | --- | --- | --- | --- | --- | --- | --- | --- | --- | --- | --- | --- |
|  |  | *N* | *28 Day (95%CI)** | *HR (95%CI)** | *Gray P** | *Adjusted HR (95%CI)* | *FG test P* | *35 Day (95%CI)* | *HR (95%CI)* | *Gray P* | *MultiHR (95%CI)* | *FG test P* |
| Age, yrs | <70 | 211 | 0.910(0.862,0.942) | Reference | 0.91 | Reference | 0.67 | 0.616(0.547,0.678) | Reference | 0.77 | Reference | 0.31 |
|  | ≥70 | 37 | 0.973(0.710,0.998) | 1.01(0.79,1.29) |  | 0.94(0.72,1.24) |  | 0.595(0.416,0.735) | 0.92(0.68,1.25) |  | 0.85(0.61,1.17) |  |
| Sex | M | 158 | 0.905(0.846,0.942) | Reference | 0.16 | Reference | 0.38 | 0.576(0.495,0.649) | Reference | 0.058 | Reference | 0.086 |
|  | F | 90 | 0.944(0.867,0.977) | 1.12(0.87,1.44) |  | 1.12(0.87,1.44) |  | 0.678(0.569,0.764) | 1.29(0.98,1.70) |  | 1.28(0.97,1.69) |  |
| KPS | ≥90 | 157 | 0.924(0.868,0.956) | Reference | 0.79 | Reference | 0.72 | 0.624(0.543,0.695) | Reference | 0.56 | Reference | 0.82 |
|  | 80 | 70 | 0.929(0.830,0.971) | 0.90(0.70,1.16) |  | 0.92(0.71,1.19) |  | 0.600(0.474,0.705) | 0.87(0.65,1.17) |  | 0.92(0.69,1.22) |  |
|  | ≤70 | 21 | 0.857(0.575,0.958) | 0.80(0.47,1.37) |  | 0.86(0.51,1.45) |  | 0.571(0.327,0.756) | 0.81(0.50,1.33) |  | 1.02(0.62,1.66) |  |
| HCTCI | 0 | 55 | 0.927(0.805,0.974) | Reference | 0.93 | Reference | 0.92 | 0.636(0.492,0.750) | Reference | 0.82 | Reference | 0.66 |
|  | 1-2 | 75 | 0.907(0.809,0.956) | 1.07(0.77,1.48) |  | 1.07(0.77,1.48) |  | 0.613(0.492,0.714) | 0.89(0.61,1.30) |  | 0.84(0.58,1.23) |  |
|  | ≥3 | 118 | 0.924(0.856,0.960) | 1.06(0.80,1.40) |  | 1.03(0.77,1.38) |  | 0.602(0.507,0.684) | 0.99(0.70,1.40) |  | 0.88(0.62,1.24) |  |
| DRI | Low-int | 186 | 0.925(0.875,0.955) | Reference | 0.84 | Reference | 0.99 | 0.613(0.539,0.679) | Reference | 0.99 | Reference | 0.31 |
|  | H/VH | 62 | 0.903(0.790,0.957) | 0.96(0.73,1.26) |  | 1.00(0.76,1.32) |  | 0.613(0.478,0.723) | 1.00(0.74,1.34) |  | 1.16(0.87,1.56) |  |
| F to M | No | 205 | 0.932(0.886,0.959) | Reference | 0.34 | Reference | 0.24 | 0.634(0.564,0.696) | Reference | 0.58 | Reference | 0.69 |
|  | Yes | 43 | 0.860(0.706,0.937) | 0.84(0.61,1.16) |  | 0.82(0.60,1.14) |  | 0.512(0.352,0.650) | 0.89(0.65,1.24) |  | 0.93(0.64,1.34) |  |
| Dr age | ≤34 | 147 | 0.918(0.859,0.953) | Reference | 0.53 | Reference | 0.26 | 0.558(0.473,0.634) | Reference | 0.75 | Reference | 0.30 |
|  | ≥35 | 101 | 0.921(0.844,0.961) | 1.09(0.85,1.39) |  | 1.16(0.89,1.51) |  | 0.693(0.592,0.774) | 1.04(0.79,1.39) |  | 1.17(0.87,1.56) |  |
| HCT era | 2015-17 | 47 | 0.851(0.703,0.929) | Reference | 0.30 | Reference | 0.44 | 0.468(0.319,0.604) | Reference | **0.020** | Reference | 0.068 |
|  | 2018-21 | 201 | 0.935(0.890,0.962) | 1.21(0.86,1.69) |  | 1.15(0.81,1.61) |  | 0.647(0.576,0.709) | 1.57(1.09,2.25) |  | 1.41(0.98,2.05) |  |
| Donor | M | 81 | 0.951(0.866,0.982) | Reference | 0.19 | Reference | 0.20 | 0.691(0.577,0.781) | Reference | **0.003** | Reference | **0.020** |
|  | MM | 49 | 0.878(0.739,0.945) | 0.89(0.62,1.29) |  | 0.89(0.62,1.29) |  | 0.673(0.520,0.787) | 0.92(0.65,1.30) |  | 0.95(0.66,1.36) |  |
|  | Haplo | 118 | 0.915(0.846,0.954) | 0.79(0.61,1.02) |  | 0.79(0.61,1.02) |  | 0.534(0.439,0.619) | 0.62(0.46,0.84) |  | 0.67(0.49,0.91) |  |

* Based on cumulative incidence curves and Gray’s test.

† Based on multivariable Fine and Gray regression models. The model for neutrophil engraftment was adjusted for donor type. The model for platelet engraftment was adjusted for HCT era and donor type.

|  | | | *Grade II-IV aGVHD* | | | | | *Grade III-IV aGVHD* | | | | |
| --- | --- | --- | --- | --- | --- | --- | --- | --- | --- | --- | --- | --- |
|  |  | *N* | *100 days (95%CI)* | *HR (95%CI)* | *Gray P* | *Adjusted HR (95%CI)* † | *FG test P*† | *100 days (95%CI)* | *HR (95%CI)* | *Gray P* | *Adjusted HR (95%CI)* † | *FG test P*† |
| Age, yrs | <70 | 211 | 0.403(0.336,0.468) | Reference | 0.71 | Reference | 0.92 | 0.161(0.115,0.214) | Reference | 0.097 | Reference | 0.16 |
|  | ≥70 | 37 | 0.351(0.202,0.505) | 0.90(0.49,1.65) |  | 0.97(0.52,1.81) |  | 0.054(0.009,0.161) | 0.32(0.08,1.37) |  | 0.35(0.08,1.53) |  |
| Sex | M | 158 | 0.405(0.328,0.481) | Reference | 0.73 | Reference | 0.55 | 0.139(0.091,0.198) | Reference | 0.68 | Reference | 0.49 |
|  | F | 90 | 0.378(0.278,0.477) | 0.94(0.62,1.41) |  | 0.88(0.58,1.33) |  | 0.156(0.089,0.238) | 1.15(0.59,2.25) |  | 1.27(0.64,2.51) |  |
| KPS | ≥90 | 157 | 0.408(0.330,0.484) | Reference | 0.83 | Reference | 0.83 | 0.134(0.086,0.192) | Reference | 0.73 | Reference | 0.97 |
|  | 80 | 70 | 0.371(0.259,0.484) | 0.87(0.56,1.36) |  | 0.87(0.56,1.36) |  | 0.157(0.083,0.252) | 1.18(0.57,2.42) |  | 1.09(0.53,2.25) |  |
|  | ≤70 | 21 | 0.381(0.177,0.584) | 0.95(0.45,2.02) |  | 1.00(0.46,2.18) |  | 0.190(0.057,0.384) | 1.50(0.51,4.42) |  | 1.04(0.36,3.00) |  |
| HCTCI | 0 | 55 | 0.364(0.238,0.490) | Reference | 0.25 | Reference | 0.32 | 0.182(0.093,0.294) | Reference | 0.51 | Reference | 0.56 |
|  | 1-2 | 75 | 0.333(0.229,0.441) | 0.95(0.53,1.70) |  | 0.96(0.54,1.71) |  | 0.107(0.050,0.189) | 0.59(0.23,1.47) |  | 0.61(0.24,1.59) |  |
|  | ≥3 | 118 | 0.449(0.357,0.537) | 1.36(0.83,2.24) |  | 1.33(0.80,2.20) |  | 0.153(0.094,0.224) | 0.85(0.40,1.81) |  | 0.90(0.42,1.95) |  |
| DRI | Low-int | 186 | 0.371(0.302,0.440) | Reference | 0.17 | Reference | 0.25 | 0.118(0.077,0.169) | Reference | **0.038** | Reference | 0.059 |
|  | H/VH | 62 | 0.468(0.339,0.587) | 1.36(0.89,2.08) |  | 1.30(0.83,2.02) |  | 0.226(0.131,0.337) | 2.02(1.04,3.92) |  | 1.86(0.98,3.53) |  |
| F to M | No | 205 | 0.405(0.337,0.471) | Reference | 0.52 | Reference | 0.63 | 0.156(0.110,0.209) | Reference | 0.27 | Reference | 0.28 |
|  | Yes | 43 | 0.349(0.210,0.492) | 0.83(0.48,1.45) |  | 0.87(0.50,1.52) |  | 0.093(0.029,0.203) | 0.57(0.20,1.57) |  | 0.56(0.19,1.60) |  |
| Dr age | ≤34 | 147 | 0.361(0.283,0.438) | Reference | 0.13 | Reference | 0.12 | 0.129(0.081,0.189) | Reference | 0.37 | Reference | 0.39 |
|  | ≥35 | 101 | 0.446(0.346,0.540) | 1.35(0.91,2.00) |  | 1.37(0.92,2.04) |  | 0.168(0.103,0.248) | 1.35(0.70,2.58) |  | 1.34(0.69,2.59) |  |
| HCT era | 2015-17 | 47 | 0.447(0.300,0.583) | Reference | 0.60 | Reference | 0.73 | 0.234(0.124,0.363) | Reference | 0.061 | Reference | 0.099 |
|  | 2018-21 | 201 | 0.383(0.316,0.450) | 0.88(0.56,1.39) |  | 0.92(0.58,1.47) |  | 0.124(0.083,0.174) | 0.51(0.25,1.03) |  | 0.57(0.29,1.11) |  |
| Donor | M | 81 | 0.346(0.244,0.450) | Reference | 0.084 | Reference | 0.079 | 0.111(0.054,0.191) | Reference | 0.18 | Reference | 0.32 |
|  | MM | 49 | 0.531(0.380,0.660) | 1.73(1.02,2.95) |  | 1.73(1.02,2.95) |  | 0.224(0.119,0.350) | 2.17(0.90,5.24) |  | 1.66(0.63,4.34) |  |
|  | Haplo | 118 | 0.373(0.286,0.460) | 1.07(0.67,1.72) |  | 1.07(0.67,1.72) |  | 0.136(0.081,0.204) | 1.23(0.54,2.76) |  | 0.94(0.39,2.24) |  |

* Based on cumulative incidence curves and Gray’s test.

† Based on multivariable Fine and Gray regression model. The model for grade II-IV aGVHD was adjusted for donor type. The model for grade III-IV aGVHD was adjusted for DRI and HCT era.

|  | | | *Any cGVHD* | | | | | *Extensive cGVHD* | | | | | |
| --- | --- | --- | --- | --- | --- | --- | --- | --- | --- | --- | --- | --- | --- |
|  |  | *N* | *1-Yr (95%CI)* | *HR (95%CI)* | *Gray P* | *Adjusted HR (95%CI)* † | *FG test P*† | *1 Yr (95%CI)* | *HR (95%CI)* | *Gray P* | *Adjusted HR (95%CI)* † | *FG test P*† |  |
| Age, yrs | <70 | 211 | 0.376(0.311,0.442) | Reference | 0.48 | Reference | 0.58 | 0.281(0.222,0.344) | Reference | 0.93 | Reference | 0.92 |  |
|  | ≥70 | 37 | 0.324(0.179,0.479) | 0.83(0.48,1.44) |  | 0.86(0.50,1.48) |  | 0.270(0.138,0.421) | 0.98(0.54,1.77) |  | 1.03(0.57,1.86) |  |  |
| Sex | M | 158 | 0.349(0.276,0.424) | Reference | 0.16 | Reference | 0.18 | 0.242(0.178,0.312) | Reference | 0.050 | Reference | 0.057 |  |
|  | F | 90 | 0.403(0.300,0.503) | 1.30(0.88,1.92) |  | 1.30(0.88,1.92) |  | 0.347(0.249,0.446) | 1.53(0.99,2.38) |  | 1.53(0.99,2.38) |  |  |
| KPS | ≥90 | 157 | 0.325(0.253,0.399) | Reference | 0.26 | Reference | 0.33 | 0.256(0.190,0.326) | Reference | 0.17 | Reference | 0.24 |  |
|  | 80 | 70 | 0.453(0.331,0.568) | 1.35(0.89,2.05) |  | 1.32(0.87,2.01) |  | 0.290(0.187,0.400) | 1.00(0.60,1.67) |  | 0.97(0.58,1.63) |  |  |
|  | ≤70 | 21 | 0.429(0.209,0.632) | 1.50(0.72,3.12) |  | 1.44(0.69,3.00) |  | 0.429(0.209,0.632) | 1.97(0.95,4.11) |  | 1.84(0.88,3.83) |  |  |
| HCTCI | 0 | 55 | 0.364(0.238,0.490) | Reference | 0.80 | Reference | 0.83 | 0.273(0.162,0.395) | Reference | 0.36 | Reference | 0.44 |  |
|  | 1-2 | 75 | 0.350(0.243,0.459) | 1.00(0.58,1.71) |  | 0.98(0.57,1.69) |  | 0.228(0.140,0.330) | 0.96(0.51,1.83) |  | 0.93(0.49,1.78) |  |  |
|  | ≥3 | 118 | 0.383(0.295,0.471) | 1.14(0.69,1.87) |  | 1.11(0.68,1.83) |  | 0.316(0.234,0.402) | 1.34(0.76,2.37) |  | 1.28(0.72,2.27) |  |  |
| DRI | Low-int | 186 | 0.367(0.297,0.436) | Reference | 0.55 | Reference | 0.70 | 0.276(0.213,0.342) | Reference | 0.77 | Reference | 0.97 |  |
|  | H/VH | 62 | 0.376(0.254,0.497) | 0.89(0.56,1.41) |  | 0.91(0.58,1.45) |  | 0.293(0.184,0.411) | 0.95(0.56,1.60) |  | 0.99(0.59,1.68) |  |  |
| F to M | No | 205 | 0.363(0.297,0.429) | Reference | 0.91 | Reference | 0.53 | 0.279(0.219,0.342) | Reference | 0.84 | Reference | 0.63 |  |
|  | Yes | 43 | 0.395(0.248,0.539) | 1.04(0.62,1.74) |  | 1.20(0.69,2.09) |  | 0.283(0.156,0.425) | 0.94(0.52,1.70) |  | 1.17(0.61,2.24) |  |  |
| Dr age | ≤34 | 147 | 0.369(0.291,0.447) | Reference | 0.92 | Reference | 0.82 | 0.267(0.198,0.341) | Reference | 0.38 | Reference | 0.31 |  |
|  | ≥35 | 101 | 0.369(0.275,0.463) | 1.04(0.70,1.53) |  | 1.05(0.71,1.55) |  | 0.299(0.212,0.390) | 1.24(0.80,1.92) |  | 1.25(0.81,1.95) |  |  |
| HCT era | 2015-17 | 47 | 0.362(0.225,0.499) | Reference | 0.82 | Reference | 0.91 | 0.213(0.108,0.340) | Reference | 0.15 | Reference | 0.28 |  |
|  | 2018-21 | 201 | 0.370(0.303,0.437) | 0.99(0.60,1.61) |  | 0.97(0.59,1.59) |  | 0.296(0.234,0.361) | 1.44(0.78,2.69) |  | 1.41(0.76,2.62) |  |  |
| Donor | M | 81 | 0.395(0.288,0.500) | Reference | 0.75 | Reference | 0.77 | 0.346(0.244,0.450) | Reference | 0.19 | Reference | 0.17 |  |
|  | MM | 49 | 0.347(0.216,0.481) | 0.93(0.54,1.59) |  | 0.88(0.51,1.52) |  | 0.245(0.134,0.373) | 0.71(0.39,1.31) |  | 0.64(0.34,1.21) |  |  |
|  | Haplo | 118 | 0.360(0.273,0.448) | 0.85(0.55,1.32) |  | 0.85(0.55,1.32) |  | 0.250(0.175,0.332) | 0.65(0.40,1.06) |  | 0.65(0.40,1.06) |  |  |

* Based on cumulative incidence curves and Gray’s test.

† Based on multivariable Fine and Gray regression models adjusted for recipient sex.
