# Supplementary material for: Melphalan-based conditioning with post-transplant cyclophosphamide for peripheral blood stem cell transplantation: donor effect
Source: Bone Marrow Transplant. 2025 Feb 27;60(5):625–31. doi: 10.1038/s41409-025-02523-3 (PMC12061766; doi:10.1038/s41409-025-02523-3)
Supplement: Supplementary file 2 — Supplementary Table 2 [file 41409_2025_2523_MOESM2_ESM.docx]

**Supplementary Table 2:** Baseline characteristics of patients with donors <35 years vs ≥35 years

|  | | **Donor Age** | | | |
| --- | --- | --- | --- | --- | --- |
|  | <35 (N=147) | | ≥35 (N=101) | Total (N=248) | p value |
| **Age at HSCT, years** |  | |  |  | 0.73 |
| Median | 63 | | 63 | 63 |  |
| Interquartile range | 57, 67 | | 58, 68 | 57, 67 |  |
| Range | (20-82) | | (23-78) | (20-82) |  |
|  |  | |  |  |  |
| **Recipient sex** |  | |  |  | 0.37 |
| Male | 97 (66%) | | 61 (60.4%) | 158 (63.7%) |  |
| Female | 50 (34%) | | 40 (39.6%) | 90 (36.3%) |  |
|  |  | |  |  |  |
| **Karnofsky performance status %** |  | |  |  | 0.16 |
| 90-100 | 99 (67.3%) | | 58 (57.4%) | 157 (63.3%) |  |
| 80 | 39 (26.5%) | | 31 (30.7%) | 70 (28.2%) |  |
| ≤70 | 9 (6.1%) | | 12 (11.9%) | 21 (8.5%) |  |
|  |  | |  |  |  |
| **HCT comorbidity index** |  | |  |  | 0.87 |
| 0 | 33 (22.4%) | | 22 (21.8%) | 55 (22.2%) |  |
| 1-2 | 46 (31.3%) | | 29 (28.7%) | 75 (30.2%) |  |
| >=3 | 68 (46.3%) | | 50 (49.5%) | 118 (47.6%) |  |
|  |  | |  |  |  |
| **Primary diagnosis** |  | |  |  | 0.64 |
| AML | 53 (36.1%) | | 37 (36.6%) | 90 (36.3%) |  |
| ALL | 24 (16.3%) | | 15 (14.9%) | 39 (15.7%) |  |
| MDS/MPN | 38 (25.9%) | | 20 (19.8%) | 58 (23.4%) |  |
| Lymphoma | 20 (13.6%) | | 20 (19.8%) | 40 (16.1%) |  |
| CML/CMMoL | 7 (4.8%) | | 7 (6.9%) | 14 (5.6%) |  |
| Others | 5 (3.4%) | | 2 (2%) | 7 (2.8%) |  |
|  |  | |  |  |  |
| **DRI** |  | |  |  | 0.33 |
| Low/intermediate | 107 (72.8%) | | 79 (78.2%) | 186 (75%) |  |
| High/very high | 40 (27.2%) | | 22 (21.8%) | 62 (25%) |  |
|  |  | |  |  |  |
| **Female donor to male recipient** |  | |  |  | 0.39 |
| Yes | 28 (19%) | | 15 (14.9%) | 43 (17.3%) |  |
| No | 119 (81%) | | 86 (85.1%) | 205 (82.7%) |  |
|  |  | |  |  |  |
| **Donor age** |  | |  |  |  |
| Median | 26 | | 41 | 32 |  |
| Interquartile range | 23, 30 | | 38, 48 | 25, 39 |  |
| Range | (11-34) | | (35-65) | (11-65) |  |
|  |  | |  |  |  |
| **Donor Type** |  | |  |  | 0.021 |
| MRD/MUD | 57 (38.8%) | | 24 (23.8%) | 81 (32.7%) |  |
| MMUD | 30 (20.4%) | | 19 (18.8%) | 49 (19.8%) |  |
| Haplo | 60 (40.8%) | | 58 (57.4%) | 118 (47.6%) |  |
|  |  | |  |  |  |
| **ABO blood group compatibility** |  | |  |  | 0.18 |
| ABO compatible | 76 (51.7%) | | 64 (63.4%) | 140 (56.5%) |  |
| Minor mismatch (donor is O) | 24 (16.3%) | | 9 (8.9%) | 33 (13.3%) |  |
| Major mismatch (Recipient is O) | 31 (21.1%) | | 16 (15.8%) | 47 (19%) |  |
| Bidirectional (None are O) | 16 (10.9%) | | 12 (11.9%) | 28 (11.3%) |  |
|  |  | |  |  |  |
| **Donor/Recipient CMV serostatus** |  | |  |  | 0.028 |
| D-/R- | 28 (19%) | | 8 (7.9%) | 36 (14.5%) |  |
| D-/R+ | 51 (34.7%) | | 32 (31.7%) | 83 (33.5%) |  |
| D+/R- | 13 (8.8%) | | 6 (5.9%) | 19 (7.7%) |  |
| D+/R+ | 54 (36.7%) | | 55 (54.5%) | 109 (44%) |  |
| Unknown | 1 (0.7%) | | 0 (0%) | 1 (0.4%) |  |
|  |  | |  |  |  |
| **Conditioning regimen** |  | |  |  | 0.014 |
| FLUDARABINE/MELPHALAN | 86 (58.5%) | | 43 (42.6%) | 129 (52%) |  |
| FLUDARABINE/MELPHALAN/TBI | 61 (41.5%) | | 58 (57.4%) | 119 (48%) |  |
|  |  | |  |  |  |
| **GVHD prophylaxis** |  | |  |  | 0.97 |
| CTX/SIROLIMUS/CELLCEPT | 10 (6.8%) | | 7 (6.9%) | 17 (6.9%) |  |
| CTX/TACROLIMUS/CELLCEPT | 137 (93.2%) | | 94 (93.1%) | 231 (93.1%) |  |
|  |  | |  |  |  |
| **HCT period** |  | |  |  | 0.053 |
| 2015-2017 | 22 (15%) | | 25 (24.8%) | 47 (19%) |  |
| 2018-2021 | 125 (85%) | | 76 (75.2%) | 201 (81%) |  |
