# Supplementary material for: Melphalan-based conditioning with post-transplant cyclophosphamide for peripheral blood stem cell transplantation: donor effect
Source: Bone Marrow Transplant. 2025 Feb 27;60(5):625–31. doi: 10.1038/s41409-025-02523-3 (PMC12061766; doi:10.1038/s41409-025-02523-3)
Supplement: Supplementary file 3 — Supplementary Table 3 [file 41409_2025_2523_MOESM3_ESM.docx]

| **Supplementary Table 3:** Incidence of hemorrhagic cystitis and infections | MRD/MUD (N=81) | MMUD (N=49) | HID (N=118) | Total (N=248) |
| --- | --- | --- | --- | --- |
| **Hemorrhagic cystitis** |  |  |  |  |
| No | 50 (61.7%) | 29 (59.2%) | 60 (50.8%) | 139 (56%) |
| Yes | 31 (38.3%) | 20 (40.8%) | 58 (49.2%) | 109 (44%) |
| *P value** |  | 0.28 |  |  |
| **Cause of hemorrhagic cystitis** |  |  |  |  |
| Chemotherapy | 25 (80.6%) | 19 (95%) | 43 (74.1%) | 87 (79.8%) |
| Viral | 6 (19.4%) | 1 (5%) | 15 (25.9%) | 22 (20.2%) |
| *P value** |  | 0.13 |  |  |
| **Hemorrhagic cystitis grade** |  |  |  |  |
| 0 | 50 (61.7%) | 29 (59.2%) | 60 (50.8%) | 139 (56%) |
| 1 | 28 (34.6%) | 19 (38.8%) | 45 (38.1%) | 92 (37.1%) |
| 2 | 3 (3.7%) | 1 (2%) | 9 (7.6%) | 13 (5.2%) |
| 3 | 0 (0%) | 0 (0%) | 4 (3.4%) | 4 (1.6%) |
| *P value** |  | 0.053 |  |  |
| **Bacterial infection** |  |  |  |  |
| No | 53 (65.4%) | 29 (59.2%) | 82 (69.5%) | 164 (66.1%) |
| Yes | 28 (34.6%) | 20 (40.8%) | 36 (30.5%) | 84 (33.9%) |
| *P value** |  | 0.43 |  |  |
| **Bacterial infection BMT CTN Grade** |  |  |  |  |
| 0 | 53 (65.4%) | 29 (59.2%) | 82 (69.5%) | 164 (66.1%) |
| 1 | 9 (11.1%) | 3 (6.1%) | 13 (11%) | 25 (10.1%) |
| 2 | 16 (19.8%) | 16 (32.7%) | 19 (16.1%) | 51 (20.6%) |
| 3 | 3 (3.7%) | 1 (2%) | 4 (3.4%) | 8 (3.2%) |
| *P value** |  | 0.42 |  |  |
| **Fungal infection** |  |  |  |  |
| No | 75 (92.6%) | 43 (87.8%) | 98 (83.1%) | 216 (87.1%) |
| Yes | 6 (7.4%) | 6 (12.2%) | 20 (16.9%) | 32 (12.9%) |
| *P value** |  | 0.14 |  |  |
| **Fungal infection BMT CTN Grade** |  |  |  |  |
| 0 | 75 (92.6%) | 43 (87.8%) | 98 (83.1%) | 216 (87.1%) |
| 1 | 6 (7.4%) | 1 (2%) | 7 (5.9%) | 14 (5.6%) |
| 2 | 0 (0%) | 1 (2%) | 3 (2.5%) | 4 (1.6%) |
| 3 | 0 (0%) | 4 (8.2%) | 10 (8.5%) | 14 (5.6%) |
| *P value** |  | 0.037 |  |  |
| **Viral infection** |  |  |  |  |
| No | 51 (63%) | 36 (73.5%) | 63 (53.4%) | 150 (60.5%) |
| Yes | 30 (37%) | 13 (26.5%) | 55 (46.6%) | 98 (39.5%) |
| *P value** |  | 0.046 |  |  |
| **Viral infection BMT CTN Grade** |  |  |  |  |
| 0 | 51 (63%) | 36 (73.5%) | 63 (53.4%) | 150 (60.5%) |
| 1 | 24 (29.6%) | 9 (18.4%) | 42 (35.6%) | 75 (30.2%) |
| 2 | 6 (7.4%) | 4 (8.2%) | 11 (9.3%) | 21 (8.5%) |
| 3 | 0 (0%) | 0 (0%) | 2 (1.7%) | 2 (0.8%) |
| *P value** |  | 0.097 |  |  |
|  |  |  |  |  |
| Any Infection |  |  |  |  |
| No | 35 (43.2%) | 21 (42.9%) | 39 (33.1%) | 95 (38.3%) |
| Yes | 46 (56.8%) | 28 (57.1%) | 79 (66.9%) | 153 (61.7%) |
| *P value** |  | 0.27 |  |  |
| Grade of any infection |  |  |  |  |
| Grade 0 | 35 (43.2%) | 21 (42.9%) | 39 (33.1%) | 95 (38.3%) |
| Grade 1 | 23 (28.4%) | 5 (10.2%) | 39 (33.1%) | 67 (27%) |
| Grade 2 | 20 (24.7%) | 18 (36.7%) | 26 (22%) | 64 (25.8%) |
| Grade 3 | 3 (3.7%) | 5 (10.2%) | 14 (11.9%) | 22 (8.9%) |
| *P value** |  | 0.15 |  |  |

* Based on chi-square or exact JT test whenever appropriate.
